# Impacted Drug-Packet in the Sigmoid Colon in a Resource-Constrained Setting: A Case Report

**DOI:** 10.1159/crg/abwag010

**Published:** 2026-09-05

**Authors:** Elroy Patrick Weledji, Verdo Zisuh Anutebeh

**Affiliations:** Department of Surgery, Faculty of Health Sciences, University of Buea, Buea, Cameroon

**Keywords:** Colorectal, Foreign body, Sigmoid colon

## Abstract

**Introduction:**

The management of a retained cannabis-filled packet in the colorectal portion of the gut depends on its depth and the consequences caused.

**Case Presentation:**

This is a case of a young male prisoner in sub-Saharan Africa, Cameroon who presented with progressive lower abdominal pain and constipation from an impacted foreign body (FB) in the sigmoid colon. This followed the insertion of the FB per annum. The primary challenges of transanal FBs which then become lodged are patient embarrassment, which can delay presentation and increase the risk of complications such as bowel perforation, and the difficulty of removing the object. Following failed conservative management, the impacted FB in the sigmoid colon was retrieved through a laparotomy.

**Conclusion:**

The learning point in this case is that a retained FB in the sigmoid colon has a much higher chance of retrieval through a laparotomy than transanally.

## Introduction

Patients with colorectal foreign bodies (FBs) continue to be seen and treated in the emergency setting with regularity in the permissive Western society. They can be from all social statuses and of all ages. Recent analyses of rectal FBs show that their incidence has been rising and most objects are inserted through the anus for sexual purposes, though ingestion, assault, auto-eroticism and body-packing are other motives. In male patients the FB is usually self-administered [1]. FBs can be classified as voluntary or involuntary and sexual or non-sexual. Involuntary cases include criminal assault, accidental insertion and body-packing for drug trafficking [2]. The novelty of this case is the manifestation of body-packing by the insertion of a cannabis-filled packet per anum to evade prison charges. In severe cases, complications such as perforation, abscess, fistula or obstruction may occur. Many patients delay seeking care due to embarrassment, leading to complications. It is important to note that FB impaction in the sigmoid colon may also occur from ingestion especially in early childhood, and even unknowingly in adults. Methods of removal of colorectal FB depend upon its location and, these are transanal, endoscopic, and operative [1, 3]. The transanal approach is successful in 60–75% of cases and the most important factor of successful removal is patient relaxation/sedation [1, 4]. The factors that determine whether a rectal FB can be removed transanally are the shape, size, location of the object, and the presence or absence of perforation [1]. Although foreign objects located in the sigmoid colon can be retrieved at the bedside, these cases are thrice more likely to require operative intervention as compared to those located more distally in the rectum [2, 4, 5]. The article was written following the CARE checklist which is attached as an online supplementary file (for all online suppl. material, see https://doi.org/10.1159/crg/abwag010).

## Case Report

A 25-year-old African male prisoner was referred as an emergency with a 5-day history of constipation associated with colicky lower abdominal pain following the insertion per annum of a pack of cigarettes refilled with dried cannabis. He had no bowel movement since, except for the intermittent passage of flatus. Physical examination revealed normal vital signs without any clinical abnormalities except for a slightly bloated abdomen with a palpable firm and immobile mass in the lower left flank. Apart from mild tenderness over the mass there was no sign of peritonitis. Rectal examination revealed a normal sphincter tone, an empty rectum, and a severe lower abdominal pain elicited on deep digital manipulation. Plain abdominal radiograph revealed a large bowel obstruction with a “cut-off” at the lower descending colon and no pneumoperitoneum (shown in Fig. 1). A rigid sigmoidoscopy to 20 cm could not reach the object. Conservative treatment with a stool softener laxative (*duphalac*) and oral fluids for 24 hrs caused more pain and discomfort with the passage of bloody mucus discharge on attempt at stooling. A decision was made for a laparotomy under general anaesthesia to retrieve the impacted FB. A further attempt at transanal extraction under general anaesthesia was not possible due to the lack of a flexible sigmoidoscope or colonoscope. Following a low midline abdominal incision, the large tense, immobile mass was seen impacted in the upper sigmoid colon. The overlying bowel was stretched and thin but not perforated (shown in Fig. 2). A proximal transverse colotomy allowed the squeezing out of the FB from the distal end (shown in Fig. 3). It measured 8 cm in length, 5 cm in width, and 6 cm in depth, and the contents were a large cylindrical packet of cigar wrappers refilled with dried cannabis and covered with plastic (shown in Fig. 4). There was no faecal contamination and the colotomy was closed in two layers: an inner interrupted sero-submucosal and an outer serosal layer continuous suturing with absorbable 3.0 vicryl. Following mass closure of the abdominal wound with non-absorbable nylon, the patient received prophylactic intravenous antibiotics for 2 days, maintenance intravenous fluids and hourly sips of water for 5 days. The bowels moved on the 5th postoperative day and, he was discharged the day after. The package would be handed over to the authority for toxicological testing if demanded. The patient was counselled on health risks and offered support. He failed to attend the follow-up appointments.

**Fig. 1.**
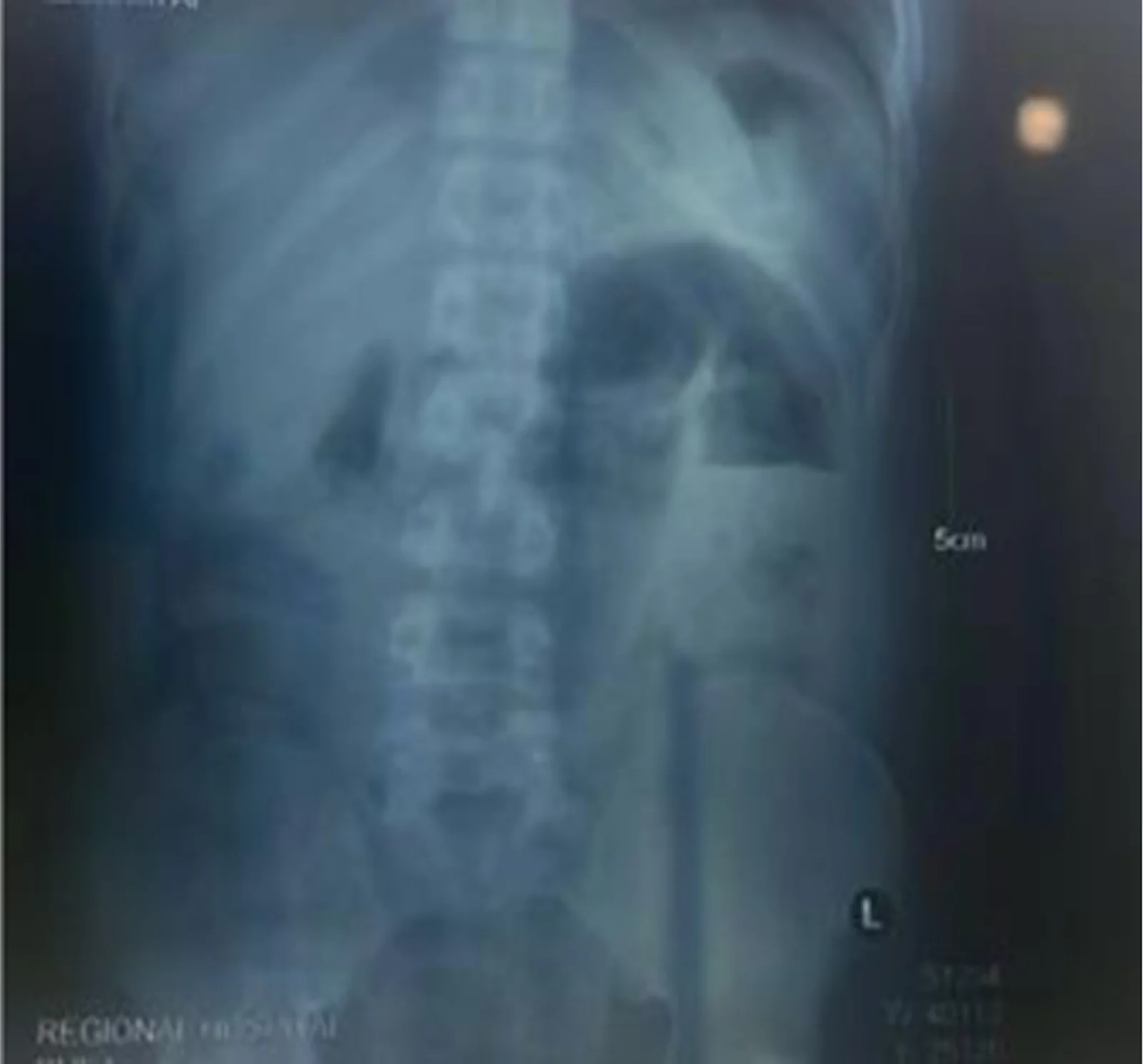
Plain Abdominal X-ray showing large bowel obstruction with a “cut-off” at descending colon.

**Fig. 2.**
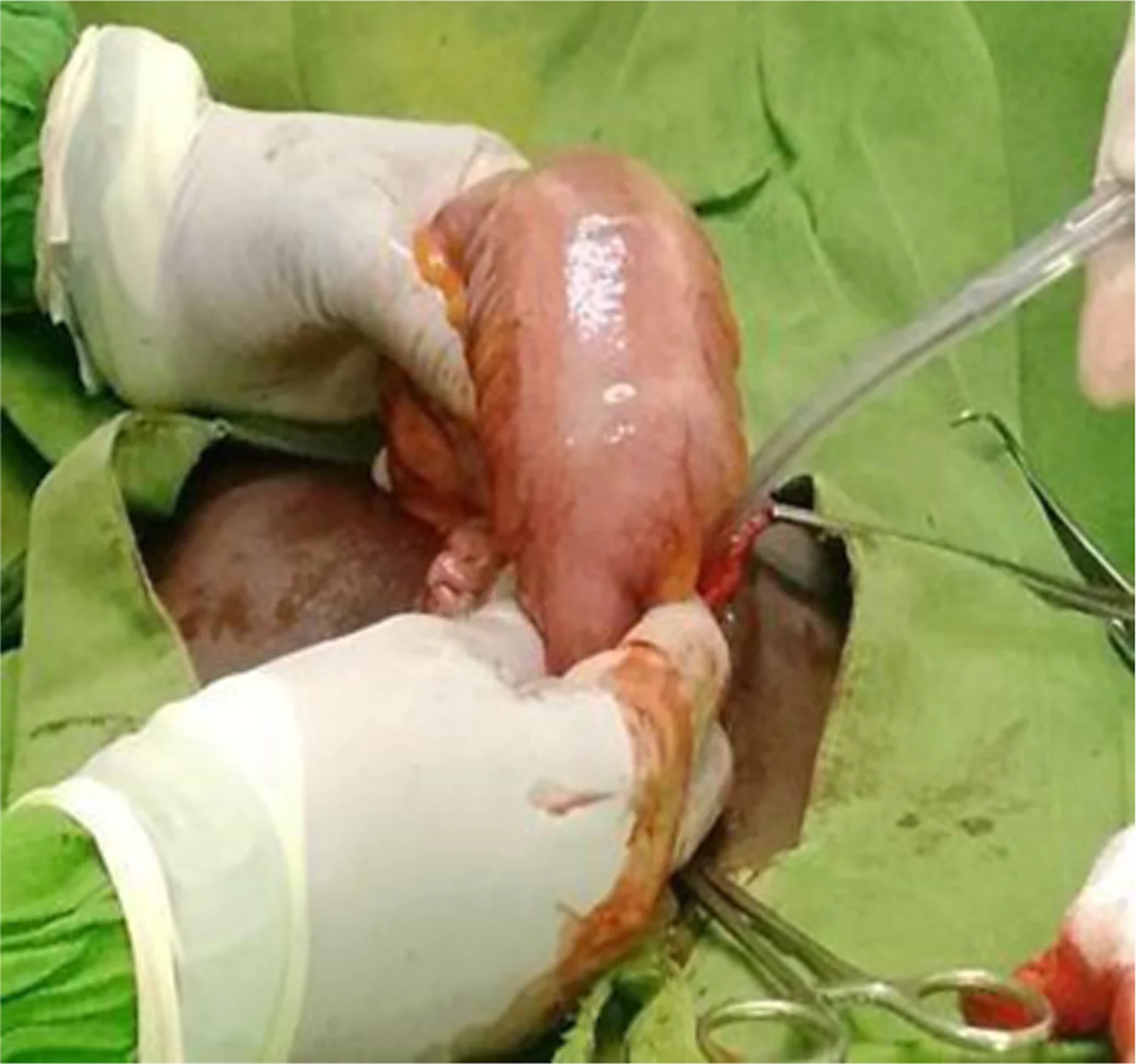
Showing impacted FB in sigmoid colon.

**Fig. 3.**
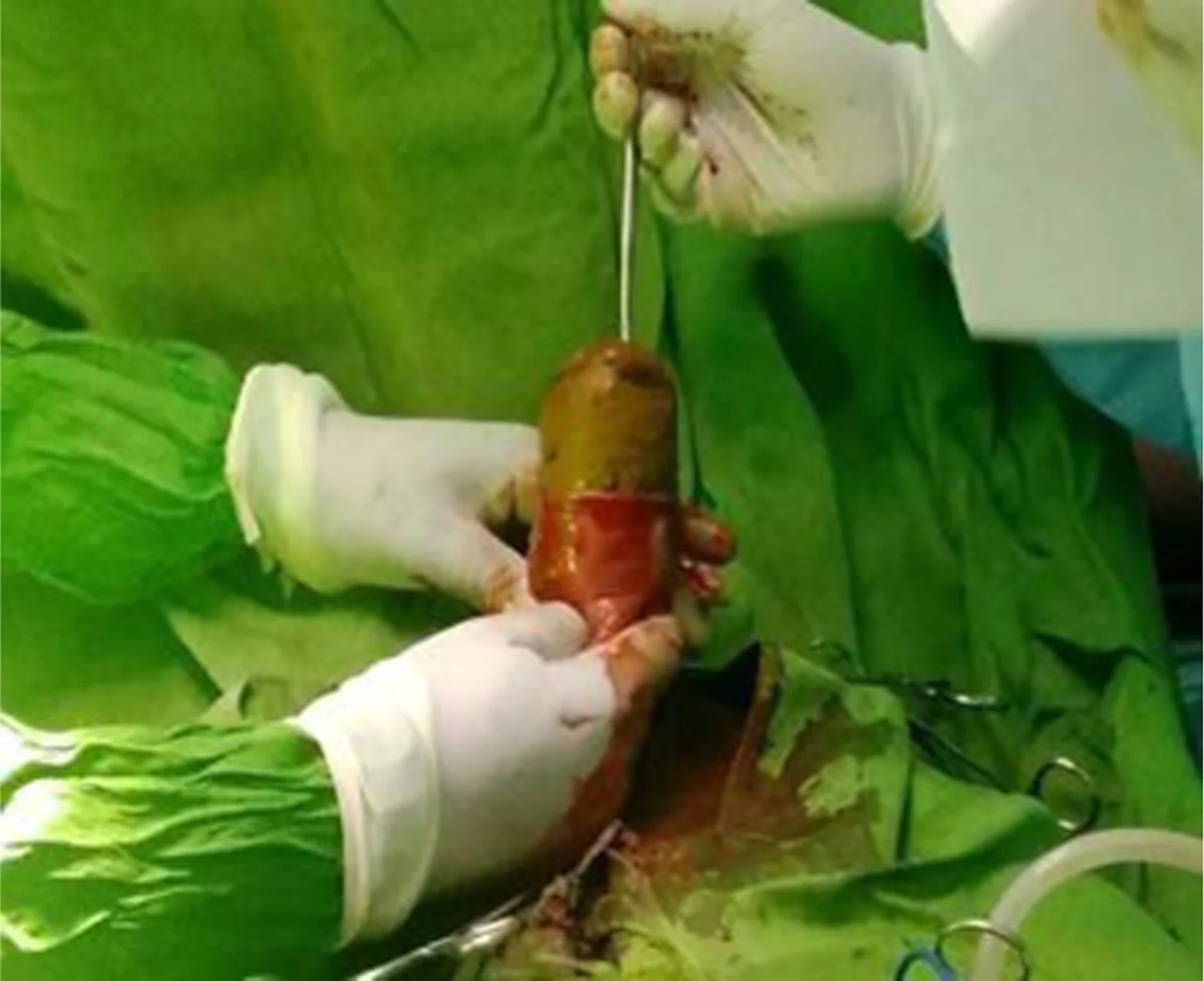
“Milking” out the FB.

**Fig. 4.**
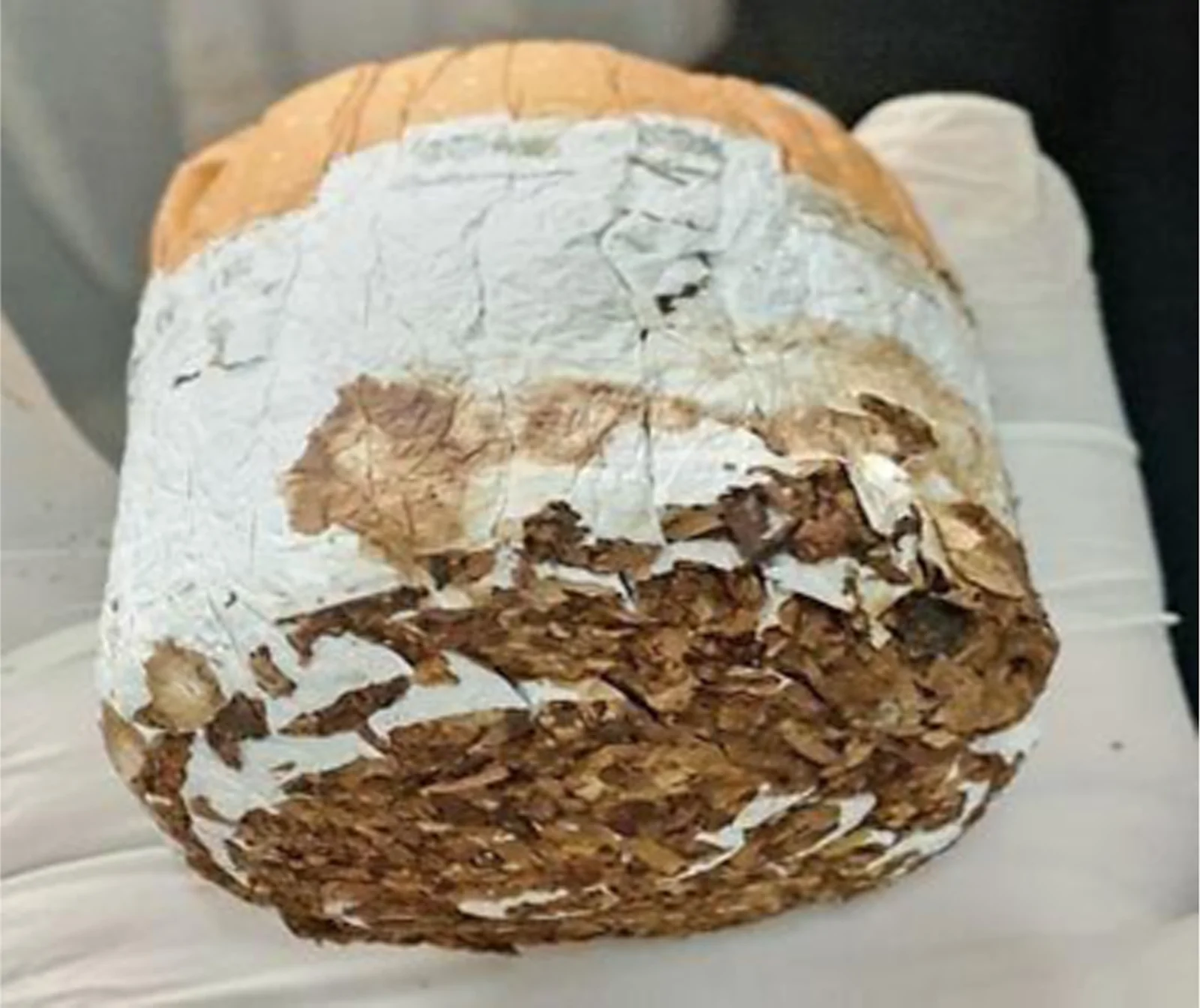
Showing FB (pack of cigar wraps refilled with cannabis).

## Discussion

Most reported cases of rectal FB impaction involve sexual stimulation or accidental insertion, whereas this case involved attempted contraband smuggling. The FB contained dried cannabis and was sealed in plastic, adding risk of rupture, and contamination. The case demonstrated the retrograde movement of a FB inserted in the rectum which later impacted in the upper sigmoid colon causing an acute large bowel obstruction. In addition to an anti-peristaltic movement of the colon, considerable oedema in the rectum may also have caused a negative suction pressure and the upward surge of the FB. The five-day delay in presentation indicates the embarrassment in relation to the patient being a prisoner and the need for a non-judgemental approach. Guidelines recommend abdominal radiography as the first-line investigation to localize the object and look for perforation or free air. Computer tomography (CT) scanning is recommended when perforation or deep impaction is suspected or when plain films are inconclusive, while ultrasound may be useful for radiolucent FBs [6]. Not performing ultrasound or CT was based on the absence of peritonitis and the clear radiographic localization. Expectant management for impacted FB in the sigmoid colon or rectum is generally not recommended. This is because of the high risk of bowel obstruction, severe ischaemia and perforation. Early controlled extraction by a colorectal specialist or gastroenterologist is the standard of care [6–8]. Non-operative management is reserved for blunt, small and stable objects (<6 cm long and 2.5 cm in diameter), visible or palpable that are expected to pass spontaneously, and, when there are no signs of peritonitis, perforation or sepsis. Close monitoring by continuous observation, serial physical examination and abdominal radiographs to track the object’s descent or identify early signs of complications is required [7]. Flexible sigmoidoscopy or colonoscopy is recommended for high-lying objects or when manual extraction fails [1, 8]. These were unavailable in our setting. The attempted rigid sigmoidoscopy reached only 20 cm, due to patient discomfort and pain from air insufflation. The presentation became a challenge because the FB was too proximal and beyond the reach of the rigid sigmoidoscopy. Secondly, the object was large with no particular sharp points to enable retrieval sigmoidoscopically. Although there were no signs of peritonitis, the object did not descend with observation and conservative treatment after 24 h and, an urgent mini-laparotomy was done to retrieve the object. At laparotomy, there was no bowel ischaemia, perforation or mesenteric laceration as a complication. As the FB was impacted, it could not be gently guided distally to come out of the anal canal and thus avoid contamination of the abdominal cavity from surgical removal. The FB was “milked” out through the colotomy on the less distended proximal colon to allow a safer primary repair. Cautery should not be used in case the FB contained an aromatic compound. There was no gross contamination requiring a diverting colostomy. Published algorithms advocate a graded approach in the management of a rectal FB. This begins with (i) initial evaluation and localization with radiographs, (ii) digital or instrument-assisted transanal extraction under sedation for low-lying objects, (iii) endoscopic removal for high-lying objects under general anaesthesia, and (iv) laparotomy for impaction, perforation or failed non-operative attempts [9]. Laparotomy was required in this case because the FB was large (8 × 5 × 6 cm), proximally located and immobile, making transanal retrieval unsafe or impossible. After failed conservative management and rigid sigmoidoscopy, laparotomy with colotomy was chosen because it offered the safest retrieval, avoiding perforation and contamination. A series reported that 3 of 15 patients with rectal FBs required laparotomy, generally for proximally located objects [10]. Of the 8 patients who underwent exploratory laparotomy from a large series of 93 cases reviewed in Los Angeles, USA, only one had succ delivery of the foreign object into the rectum for transanal extraction. The remainder required repair of perforated bowel or retrieval of the FB via a colotomy [11]. These data support the assertion that impacted sigmoid objects often necessitate surgery. An alternative method would have been a colonoscopy using gastrolith forceps or endoscopic snare or if room available inserting a few Foley catheters (20–22 Fr) alongside the object and pumping air into the rectum and lower sigmoid colon [1, 8, 12, 13]. The latter will break the negative suction and when the Foley catheter balloons are inflated with 30-50 mLs of saline and gently pulled back it should bring the object down to the rectum to be retrieved. These alternative minimally invasive methods, such as endoscopic snare removal, gastrolith forceps or Foley catheter balloon extraction were not feasible in this case given the object’s size, shape and location. Early Imaging with radiography or computed tomography is often necessary to locate the object and assess complications. A Transanal Minimally Invasive Surgery (TAMIS) platform provides a viable alternative when endoscopic retrieval is unsuccessful or the FB is located high and firmly impacted [1, 8]. Potential complications after retrieval of the FB regardless of the method include perforation, bleeding, infection, and pressure necrosis. Broad-spectrum antibiotics, careful handling and avoidance of electrocautery (to prevent explosions when aromatic compounds are present) were used. CT or ultrasound can detect perforation or abscesses when suspected [8, 14]. A rigid or flexible sigmoidoscopy should be performed to rule out any obvious laceration, haematoma, or other traumatic injury to the rectum and lower colon [1–3, 8]. The learning point in this case is that a retained FB in the sigmoid colon has a much higher chance of retrieval through a laparotomy than transanally.

Even after operative retrieval, a postoperative endoscopic assessment or repeat imaging ensures no retained fragments and that healing is adequate [8, 14]. Post extraction evaluation was a limitation in this case. Patients with rectal FBs may experience shame, anxiety or underlying psychiatric issues. A non-judgemental approach improves disclosure and reduces delays. There is the need for psychological evaluation and counselling, especially for individuals engaging in high-risk behaviours or drug smuggling [15].

## Conclusion

This case demonstrated the unusual presentation of an impacted drug packet in the sigmoid colon causing intestinal obstruction which required an operative intervention. Medical management involves a step-wise approach, from manual extraction to surgical intervention, and often requires addressing potential psychological issues associated with the incident.

## Statement of Ethics

Ethics approval was not required for this study in accordance with local or national guidelines. Written informed consent was obtained from the patient for publication of this case report and any accompanying images.

## Conflict of Interest Statement

The authors have no conflicts of interest to declare.

## Funding Sources

This study was not supported by any sponsor or funder.

## Author Contributions

E.P.W.: data curation, formal analysis, methodology, resources, writing – original draft, and writing – review and editing. V.Z.A.: resources and review.

## Data Availability

The data that support the findings of this study are available on request from the corresponding author. The data are not publicly available due to privacy or ethical restrictions.
